# Case Report: Hemophagocytic Lymphocytosis in a Patient With Glutaric Aciduria Type IIC

**DOI:** 10.3389/fimmu.2021.810677

**Published:** 2022-01-13

**Authors:** Lingtong Huang, Wei Wu, Yijing Zhu, Huili Yu, Lingling Tang, Xueling Fang

**Affiliations:** ^1^ Department of Critical Care Units, the First Affiliated Hospital, Zhejiang University School of Medicine, Hangzhou, China; ^2^ Department of Infectious Diseases, the First Affiliated Hospital, Zhejiang University School of Medicine, Hangzhou, China; ^3^ Department of Hematology, the First Affiliated Hospital, Zhejiang University School of Medicine, Hangzhou, China; ^4^ Department of Infectious Diseases, Shulan (Hangzhou) Hospital, Zhejiang Shuren University of Shulan International Medical College, Hangzhou, China

**Keywords:** glutaric aciduria, hemophagocytic lymphocytosis, hemophagocytic syndrome, cytopenia, inborn errors of metabolism, IEM

## Abstract

Hemophagocytic lymphocytosis (HLH) is a rare disease caused by inborn errors of immunity (IEI), secondary to infection, lymphoma or autoimmune disorders, but we often overlook the fact that HLH can be secondary to inborn errors of metabolism (IEM). Here, we describe a patient who was diagnosed with glutaric aciduria type IIC complicated by features suggestive of possible HLH. The diagnosis of glutaric aciduria type IIC, a IEM, was confirmed by whole exome sequencing. The patient was treated with coenzyme Q10 and riboflavin which effectively improved her liver function. During treatment, the patient developed severe anemia and thrombocytopenia. Persistent fever, splenomegaly, cytopenias, increased ferritin, hypertriglyceridemia, hypofibrinogenemia, and hemophagocytosis in the bone marrow pointed to the diagnosis of HLH; however, the patient eventually died of gastrointestinal bleeding. After other potential causes were ruled out, the patient was diagnosed with glutaric aciduria type IIC complicated by features suggestive of possible HLH. When cytopenias occurs in IEM patients, HLH is a possible complication that cannot be ignored. This case suggests a possible relationship between IEM and risk for immune dysregulation.

## Introduction

Hemophagocytic lymphocytosis (HLH) is a rare fatal disease with extremely high mortality rates. It often results from genetic defects in immune system function or due to infections (such as Epstein-Barr virus, Cytomegalovirus, Parvovirus B19), tumors, and autoimmune disorders. HLH may also be caused by inborn errors of metabolism (IEM), a trigger which may often be overlooked (1). Here, we describe an adult with glutaric aciduria type IIC, a IEM, who developed features suggestive of HLH during the diagnosis and treatment of their underlying disease.

Glutaric aciduria is a systemic disease caused by errors in fatty acid oxidation and function of several mitochondrial dehydrogenase enzymes (2). In most cases, this condition has a childhood onset; however, some cases of adulthood onset disease have been reported, possibly due to late-onset multiple acyl-CoA dehydrogenase deficiency (3). Most patients develop neurological symptoms at the onset of illness (4), accompanied by repeated hypoglycemia (5), hyperlactic acidemia, and hyperlipidemia.

To our knowledge, this is the first case of glutaric aciduria type IIC complicated by HLH. Moreover, this case underscores the importance of considering HLH in patient with IEM and signs as well as symptoms of immune dysregulation. This case also provides evidence for the potential link between IEM and immune dysregulation.

## Method

### Whole Exome Sequencing

The genomic DNA was randomly broken into fragments with a length of 180-280 bp by a Covaris breaker. After end repair and A-tailing, the two ends of the fragment were ligated with adapters to prepare a DNA library. The library with a specific index was pooled with up to 500,000 biotin-labeled probes for liquid phase hybridization, and then the n exons of n genes were captured by magnetic beads with streptomycin, and linearly amplified by PCR. After the increase, the library quality inspection was carried out, and the sequencing could be carried out if it was qualified. After the library was constructed, Qubit 2.0 was used for preliminary quantification, and then Agilent 2100 was used to detect the insert size of the library. After the insert size meet expectations, qPCR was used to accurately quantify the effective concentration (3nM) of the library to ensure the library quality. The library was qualified, and the Illumina platform was used for sequencing according to the effective concentration of the library and the data output requirements. AfterQC was used to evaluate the sequencing quality of the off-machine original sequencing data, and removed low-quality and contaminated reads. The filtered data was sequenced with the human hg19 reference genome using BWA software (Burrows Wheeler Aligner), and then the capture effect was evaluated. GATK software (Genome Analysis Toolkit) was used to analyze SNV (single nucletide variant) and Inde (linsertion and deletion) in the genome. Then the population database 1000 Genomes (1000 human genome dataset), Genome AD (Genome Aggregation Database dataset) 2.1.1 and ExAC (The Exome Aggregation Consortium dataset) was used to filter the analyzed SNV and Indel. The dbNSFP database was used to predict the pathogenicity of missense mutations and splicing mutations. Human Mendelian Inheritance Database (OMIM), Human Gene Mutation Database (HGMD) and Clinvar Database was used to screen for reported mutations. Finally, Sanger sequencing was used to verify all possible pathogenic sites.

### Literature Search

A literature search was conducted on PubMed, using the keywords “glutaric aciduria” for case reports and case series written before December 2021 to assess whether this is the first case report of glutaric aciduria type IIC complicated by HLH. Another literature search was conducted on PubMed, using (Inborn errors of metabolism) AND (Hemophagocytic) for case reports and case series written before December 2021 to summarize the cases of IEM complicated by HLH. It should be noted that we did not conduct meta-analysis and systematic reviews, but only reviewed the literature that was queried.

### Case Report

A 27-year-old woman had persistent weakness in her upper and lower limbs for 10 years. The weakness of her upper and lower limbs did not affect her work and life. She was misdiagnosed with seronegative polymyositis for which she received 2.5 milligrams prednisone per day one year prior to admission. In the month prior to admission, she gradually became unable to take care of herself. She had no other previous medical history, nor had she traveled abroad. She was not pregnant. The patient’s parents were healthy as was her younger brother and son. There was no genetic disease in her family. Her physical examination was normal except for weakness of the upper and lower limbs.

**Figure 1:**
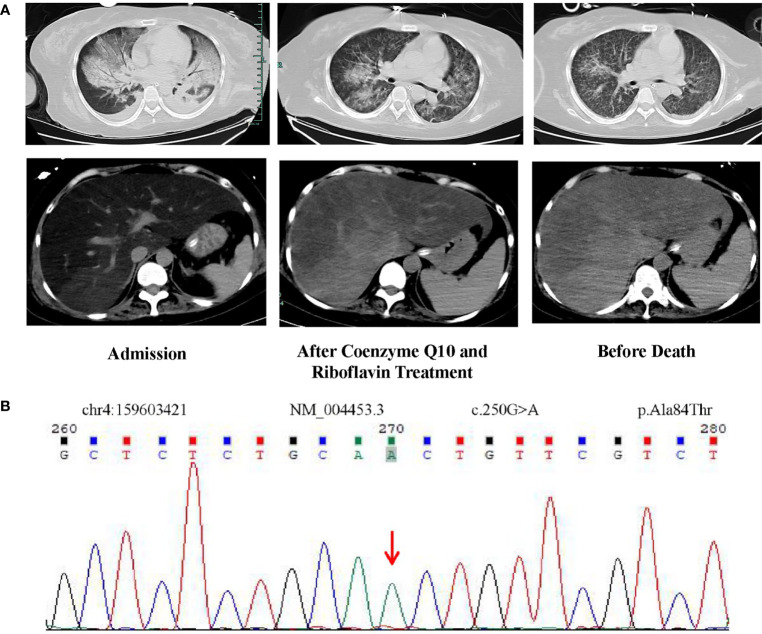
**(A)** CT images of the lungs and abdomen on admission, after coenzyme Q10 and riboflavin treatment, and before death. After treatment with riboflavin and coenzyme Q10, the CT value of the patient’s liver gradually increased from -40 Hu at the beginning to nearly normal. **(B)** Homozygous mutation of *ETFDH* gene (c.250G>A) identified by whole exome sequencing.

Following admission, she developed repeated episodes of hypoglycemia, hyper lactic acidemia, and hyperlipidemia (**Table 1**). On the fifth day, she was transferred to critical care unit due to respiratory failure, anuria, and liver failure. Both lungs showed large patchy lesions, and the density of the liver was quantitatively measured by CT image as -40 Hu, which was lower than the density of water (0 Hu) and was similar to the density of fat (-40 Hu) (**Figure 1A**). Since the patient showed persistent fever, next-generation sequencing for infectious pathogens and culture of bronchoalveolar lavage fluid were performed to rule out infectious pathogens such as Cytomegalovirus, Herpes simplex virus, Epstein-Barr virus and Pneumocystis in the respiratory tract. The method of mNGS was the same as discribed before (6).

**Table 1:** Laboratory Data.

Variable	Reference Range	Admission	In ICU HD5	HD15	HD31	Before Death
Hematocrit (%)	38-50.8	43.8	44.2	24.5	12.7	21.7
Hemoglobin (g/dl)	13.1-17.2	14.8	15.2	8.0	4.5	6.3
Platelet count (10^9/L)	83-303	366	314	198	9	270
Red-cell count (10^12/L)	4.09-5.74	4.83	4.94	2.68	1.49	2.3
Mean corpuscular volume (fl)	83.9-99.1	90.7	88.9	91.2	91.4	94.3
Fibrinogen (g/L)	2.0-4.0	1.29	1.23	2.56	2.31	1.33
Activated partial-thromboplastin time (sec)	23.9-33.5	30.4	>150	33.9	34.2	40.7
Alanine aminotransferase (U/L)	9-50	114	179	357	61	20
Aspartate aminotransferase (U/L)	15-40	473	700	636	152	61
Lactate dehydrogenase (U/L)	120-250	ND	2596	1819	ND	ND
Total Cholesterol (mmol/L)	3.14-5.86	10.71	ND	6.83	ND	ND
Triglycerides (mmol/L)	0.3-1.7	8.43	ND	4.28	ND	ND
Lactic acid (mmol/L)	0.5-2.2	3.7	4.3	0.9	1	3.5
Ammonia (μmol/L)	10-47	70	165	98	36	ND
fasting blood-glucose (mmol/L)	3.9-6.1	1.42	4.3	0.5	6.1	6.3
SOFA score			21			9
Apache II score			26			13

HD, hospitalization day; ND, Not done.

As the patient had hypoglycemia, diagnosis of glycogen storage disease was considered. Due to the patient’s persistently abnormal coagulation parameters (**Table 1**), a liver biopsy was not performed. A muscle biopsy did not demonstrate obvious lipid deposits, but magnetic resonance imaging (MRI) of the lower limbs revealed a large amount of fat accumulation between the muscles. The patient remained aurinc due to renal failure, so the urine organic acids were not performed. Results of whole exome sequencing revealed a homozygous mutation of *ETFDH* gene (c.250G>A) as shown in **Figure 1B**. The patient was diagnosed with glutaric aciduria type IIC and was treated with 150mg riboflavin per day and 40mg coenzyme Q10 per day. The patient was alos given a high-sugar and low-fat diet. CT imaging suggested that the patient’s liver was improved significantly which was confirmed by laboratory tests (**Figure 3**). Despite this improvement, the patient developed severe cytopenias (45g/L of hemoglobin and 9×10^9^/L of platelets).

Severe cytopenias are not typically seen in patients with glutaric aciduria type IIC. Other complicating diagnoses which could explain the findings of cytopenias in glutaric aciduria type IIC were considered. The patient developed acute renal failure with anuria shortly after admission suggesting consideration of thrombotic microangiopathy. Peripheral blood smear findings of thrombotic microangiopathy including mechanical haemolytic anaemia were not found, and pathogenic mutations in genes such as *CD46, CFI, CFB, C3, THBD* and *CFH* were absent (7). Acute fatty liver of pregnancy (AFLP) or hemolysis, elevated liver enzymes, and low platelets (HELLP) were also considered which may occur in pregnant women (8), and in pregnant women with IEM (8, 9); however, the possibility of pregnancy was excluded. NGS and cultures were also performed on samples of bronchoalveolar lavage fluid, peripheral blood, and peritoneal fluid, however, no pathogen was isolated which suggested the presence of aseptic inflammation. The persistent and severe hyperlipidemia suggested oxidative stress induced hemolysis; however, peripheral blood smear findings of oxidative hemolysis such as G6PD deficiency were absent. Similarly, whole exome sequencing did not document any pathogenic of G6PD enzyme deficiency-related gene mutations.

**Figure 2:**
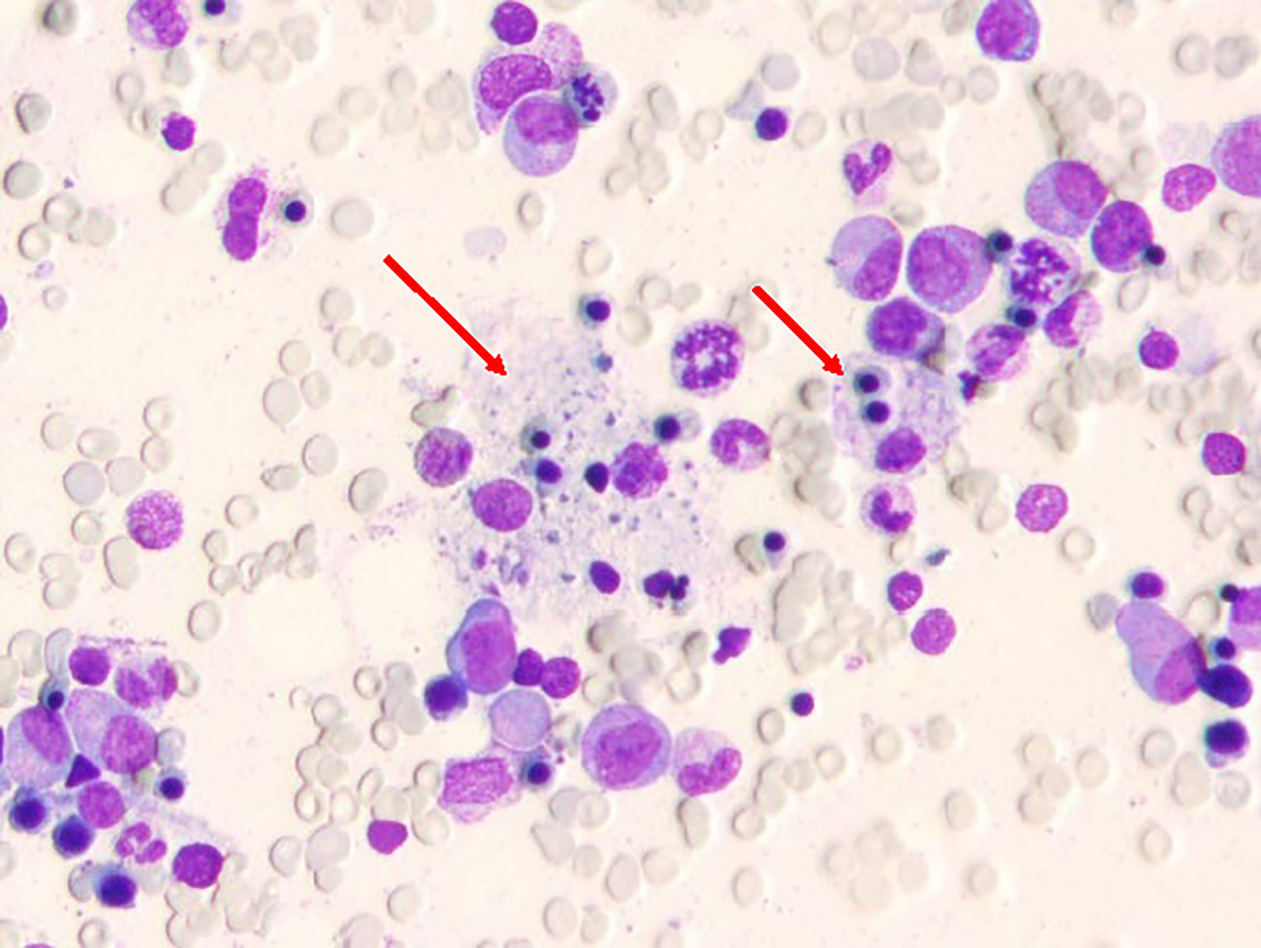
Bone marrow smear carried out when hemoglobin was lowest; the arrow indicates hemophagocytic cells.

**Figure 3:**
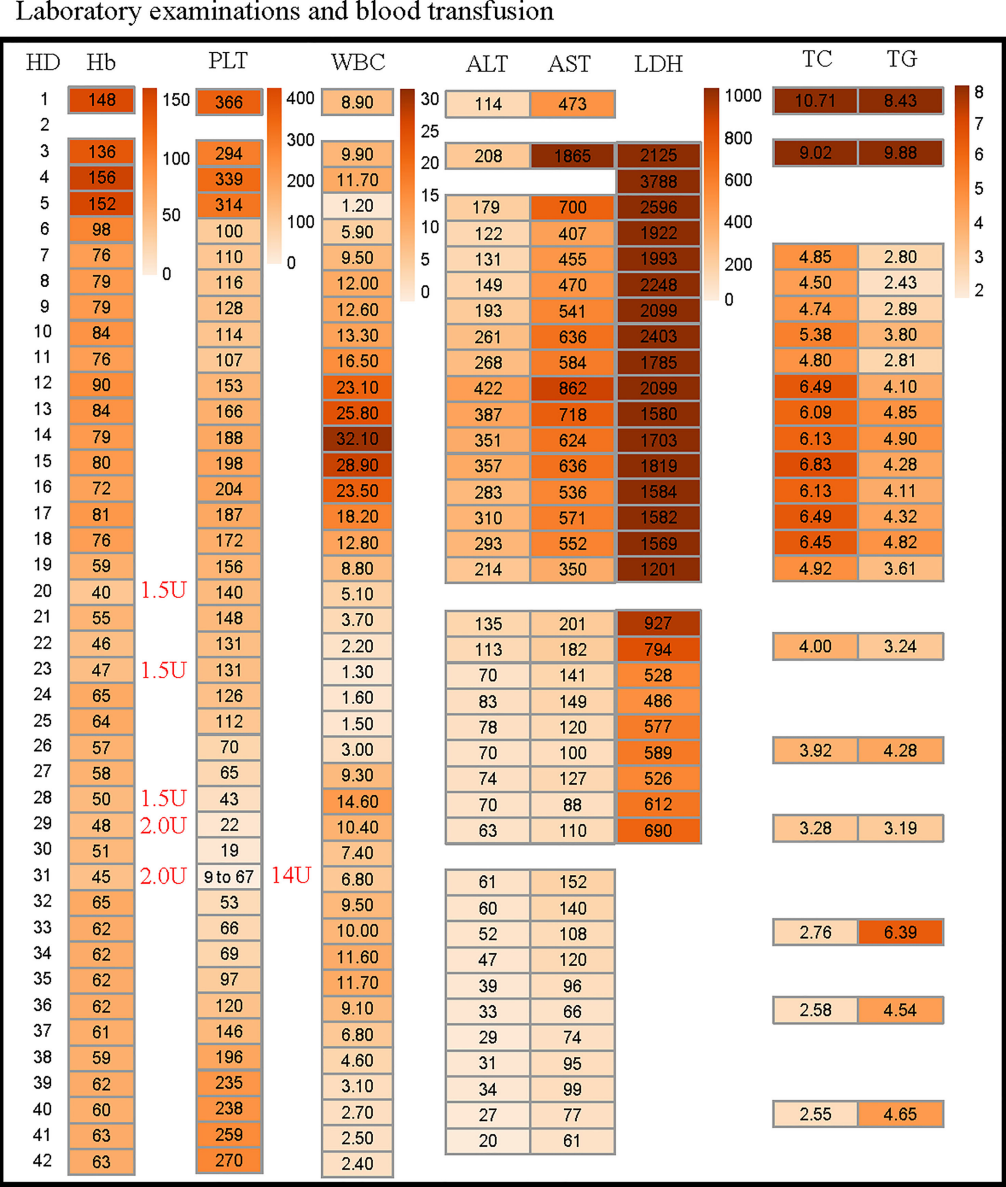
Changes in patient’s blood routine, liver function within 42 days after admission. The reference range for each variable are shown in **Table 1**. The amount of red blood cells and platelet administered to the patient are marked with red font on the right side of the corresponding time. HD, hospitalization day; Hb, hemoglobin; PLT, platelets; WBC, white blood cell; ALT, alanine aminotransferase; AST, aspartate aminotransferase; LDH, lactate dehydrogenase; TC, total cholesterol; TG, triglycerides.

Finally, hemophagocytosis was observed on a bone marrow biopsy and aspiration. There are eight diagnostic criteria for hemophagocytic lymphocytosis (1), and the patient met six of them, including persistent fever, splenomegaly (**Figure 1A**), cytopenias (**Figure 3**), increased ferritin (1000 ng/mL, reference range 7-323 ng/mL), hypertriglyceridemia and hypofibrinogenemia (**Table 1** and **Figure 3**), and hemophagocytosis in the bone marrow (**Figure 2**). Of note, serum soluble IL-2R and NK cell activity were not tested in this case. Combined with these laboratory tests, the diagnosis of HLH was suggested. Results from whole exome sequencing showed no gene mutation such as PRF1, UNC13D, STXBP2, STX1, RAB27A, LYST, AP3B1, SH2D1A or XIAP which implied that there was no primary HLH. Besides, no evidence of malignancy, infections, or autoimmune disorders were found. Therefore, we attributed the cause of HLH features to glutaric aciduria type IIC. Considering that this patient had a clear trigger, other treatments (e.g. etoposide, steroids, cyclosporine) were not administered. Supportive care including infusion of red blood cells was performed. Her hemoglobin was maintained at 60g/L, and her platelets gradually increased from 9×10^9/L to normal after the day 32 of hospitalization as liver function continued to improve (**Figure 3**). Unfortunately, she eventually died of gastrointestinal bleeding despite remission of the features of HLH after being hospitalized for a month and a half. The patient’s family declined an autopsy.

## Discussion

Abnormal blood biochemical examinations such as lactic acid, blood glucose and blood lipids in adults can often lead clinicians to consider the diagnosis of a IEM. In addition to biochemical examinations, whole exome sequencing has aided in the rapid diagnosis of IEM. In this case, the patient showed no obvious neurological symptoms except for upper and lower extremities weakness. The patient’s condition progressed to severe hypoglycemia and hyperlipidemia, which is consistent with the clinical manifestations of glutaric aciduria type II. In the east of China, homozygous mutation of *ETFDH* gene (c.250G>A) is the most common cause of glutaric aciduria type IIC (10, 11). This genetic mutation was found in this case (**Figure 1B**). Given that the CT appearance of liver (**Figure 1A**), lipid deposition was suspected. The patient was eventually diagnosed with glutaric aciduria type IIC. Consequently, the patient was administered a high-dose coenzyme Q10 and riboflavin—the two drugs recommended for the disease (2) and the clinical manifestations improved rapidly.

Few people would consider HLH in the differential diagnosis of cytopenias in IEM patients. She had very serious liver damage and hypertriglyceridemia. She also had multi organ failure, including anuria, respiratory failure and liver failure, which made it easy to overlook the HLH features. As a critically ill patient, all of her clinical symptoms were non-specific. After considering and excluding important diagnoses associated with acute onset of cytopenias, the diagnosis of HLH was considered. HLH is a fatal disease which is often caused by genetic defects, or it may develop secondary to malignancy, autoimmune diseases, and infections (1). However, none of these factors were found during the disease course of our patient. Therefore, we attributed the occurrence of HLH to glutaric aciduria type IIC.

In the past 30 years, cases of IEM complicated by HLH have been reported (**Table 2**). Almost all cases occured in children, so in the treatment of adult IEM patients, the diagnosis of HLH may be overlooked. Some reported cases were associated with Lysosomal Storage Disease (LSD), including Gaucher Disease (GD) (21, 22), Chediak-Higashi Syndrome (CHS) (29), Griscelli’s Disease (28), Hermansky-Pudlak Syndrome Type II (HPSII) (23, 24), Wolman’s Disease (a type of lysosomal acid lipase deficiency) (25–27). NK cell dysfunction could be found in some LSDs (e.g. CHS, Griscelli’s Disease, HPSII) because of lysosomal dysfunction, so it is also classified as IEI. Excluding LSD, many forms of IEM can lead to the occurrence of HLH. Disorders of lipid metabolism such as glutaric aciduria type IIC and long-chain 3-hydroxyacyl-CoA dehydrogenase deficiency (13) or disorders of organic acid metabolism such as lysinuric protein intolerance (LPI) (19, 20, 35), methylmalonic acidemia (17), propionic acidemia (17, 18) may be complicated by HLH. There are many other rare IEMs complicated by HLH that are also reported such as biotinidase deficiency (12), hepatolenticular degeneration (33), mevalonate kinase deficiency (30, 31), pyrimidine deficiency (32), disorder of glycogen metabolism (14, 15), prolidase deficiency (16) and cobalamin C disease (34).

**Table 2:** Reported cases of IEM complicated by HLH.

Reference	Type of IEM	Age at onset of HLH features	Concomitant symptoms in addition to HLH features	Treatment for HLH	Treatment responses	Prognosis
***Disorder of energy metabolism***						
This paper	*Glutaric Aciduria Type IIC*	27-year-old	hypoglycemia and metabolic acidosis	no treatment	remission	died
Kardas et al. (12)	*Biotinidase Deficiency*	4-month-old	/	IVIG	remission	alive
Erdol et al. (13)	*Long-chain 3-hydroxyacyl-CoA Dehydrogenase Deficiency*	4-month-old	/	IVIG; PE	lack of remission	died
Düzenli et al. (14)	*Type Ia Glycogen Storage Disease*	5-month-old	hypoglycemia	HLH-2004 protocol	remission	alive
Wei et al. (15)	*Type IV Glycogen Storage Disease*	11-month-old	/	dexamethasone; ruxolitinib	remission	/
***Disorder of organic acid metabolism***						
Rossignol et al. (16)	*Prolidase Deficiency*	all child	/	IVIG, corticoids, and ganciclovir for one confirmed case; cyclosporine and dexametha for one suspected case	patient 1 was remission; patient 2 was lack of remission	/
Gokce et al. (17)	*Methylmalonic Acidemia*	4-year-old	metabolic acidosis and deterioration of consciousness	HLH-2004 protocol; PE	lack of remission	died
Gokce et al. (17)	*Propionic Acidemia*	patient 1 was 2-year-old; patient 2 was 7-year-old	Both patients showed metabolic acidosis and deterioration of consciousness	patient 1 received HLH-2004 protocol and PE; patient 2 received IVIG and cyclosporine	all remission	alive
Aydin et al. (18)	*Propionic Acidemia*	2-month-old	deterioration of consciousness	IVIG and HLH-2004 protocol	remission	alive
Duval et al. (19)	*Lysinuric Protein Intolerance*	all child	/	/	/	/
Ouederni et al. (20)	*Lysinuric Protein Intolerance*	9-month-old	gastrointestinal symptoms	no treatment	remission	alive
***Lysosomal storage disease (LSD)***						
Sharpe et al. (21)	*Gaucher Disease*	newborn	/	HLH-2004 protocol; HSCT	lack of remission	died
Schüller et al. (22)	*Gaucher Disease*	newborn	/	/	/	/
Enders et al. (23)	*Hermansky-Pudlak Syndrome Type II*	2-year-old	severe bleeding episode	/	/	died
Dell’Acqua et al. (24)	*Hermansky-Pudlak Syndrome Type II*	17-year-old	/	dexamethasone; etoposide	lack of remission	died
Essa et al. (25)	*Wolman's Disease (a type of lysosomal acid lipase deficiency)*	from 2-month-old to 4-month-old	Both patients showed severe gastrointestinal symptoms	/	/	all died
Taurisano et al. (26)	*Wolman's Disease*	4-year-old	severe gastrointestinal symptoms	/	/	died
Rabah et al. (27)	*Wolman's Disease*	2-month-old	severe gastrointestinal symptoms	HLH-2004 protocol	lack of remission	died
Goldberg et al. (28)	*Griscelli's Disease*	all juvenile	/	/	/	/
Rubin et al. (29)	*Chediak-Higashi Syndrome*	11-month-old	/	methylprednisolone; HSCT	lack of remission	died
***Other IEM***						
Rigante et al. (30)	*Mevalonate Kinase Deficiency*	7-year-old	arthralgias and deterioration of consciousness	methylprednisolone and cyclosporine	remission	alive
Tanaka et al. (31)	*Mevalonate Kinase Deficiency*	all child	/	one patient received the HLH-94 protocol and HSCT; the other one received repeated PE	/	patient 1 died ; patient 2 alive
Pérez-Torras et al. (32)	*Pyrimidine Deficiency*	2-month-old	metabolic acidosis	/	lack of remission	died
Yokoyama er al (33).	*Hepatolenticular Degeneration*	10-year-old	/	methylprednisolone; cyclosporine A; PE	remission after liver transplantation	alive
Wu et al. (34)	*Cobalamin C Disease*	4-month-old	increased creatinine	no treatment	remission	alive

IVIG, Intravenous immunoglobulin; HSCT, hematopoietic stem cell transplantation; PE, plasma exchange; /, not mentioned.

These signs and symptoms of HLH occurring in the context of IEM indicate that different IEM complicated by HLH have heterogeneity. In addition to the typical symptoms of HLH, most IEM patients with HLH also have many clinical manifestations that may be related to the primary disease. Some patients may develop metabolic encephalopathy (17, 18, 30), and some patients may have severe gastrointestinal symptoms (20, 26, 27). Metabolic acidosis is also a relatively common clinical manifestation in IEM complicated by HLH (17, 32). Treatments may be variable and may include IVIG, etoposide, cyclosporine, plasma exchange and hematopoietic cell transplantation (table 2). Non-LSD IEM patients may not need targeted treatment of HLH features and signs of HLH may regress as the primary disease improves (19, 20, 34, 35). For the treatment of IEM complicated by HLH, we recommend that the patient’s HLH features be carefully monitored with respect to the response of the treatment of the underlying IEM. In this case, although the patient eventually died of gastrointestinal bleeding, the patient responded well to riboflavin and coenzyme Q10, and her HLH features showed signs of remission which suggests that in patients with IEM, the treatment of the primary disease may be crucial. However, it should be noted that the treatment of such patients still requires the cooperation of metabolic physicians, immunologists, hematologists and intensive care physicians to develop an individualized treatment plan.

However, we cannot clarify the causal relationship between glutaric aciduria type IIC and HLH and we have not explored the pathogenesis which are the limitations of this case report. The rare incidence of IEM and the rare complication of HLH limit the ability to often consider the diagnosis of HLH when faced with a patient with a IEM. Also, due to insufficient knowledge of the potential association of IEM and HLH, many patients may be misdiagnosed. Many potential links between metabolism and immunity have been discovered (36, 37). This case provides evidence for the relationship between IEM and impaired immune function. When cytopenias occur in IEM patients, HLH is a possible complication that cannot be ignored.

## Data Availability Statement

The raw data supporting the conclusions of this article will be made available by the authors, without undue reservation.

## Ethics Statement

The studies involving human participants were reviewed and approved by the ethics committee of the First Affiliated Hospital of Zhejiang University. The patients/participants provided their written informed consent to participate in this study. Written informed consent was obtained from the individual(s) for the publication of any potentially identifiable images or data included in this article.

## Author Contributions

XF, LH, WW, YZ, HY wrote the first draft made the initial diagnosis. XF, LH, LT participate in the discussion of the diagnosis. We all and cared for the patient and reviewed the final manuscript.

## Funding

This work was supported by the National Natural Science Foundation of China grant 81872672 (to LT).

## Conflict of Interest

The authors declare that the research was conducted in the absence of any commercial or financial relationships that could be construed as a potential conflict of interest.

## Publisher’s Note

All claims expressed in this article are solely those of the authors and do not necessarily represent those of their affiliated organizations, or those of the publisher, the editors and the reviewers. Any product that may be evaluated in this article, or claim that may be made by its manufacturer, is not guaranteed or endorsed by the publisher.
