# Supplementary figures and images for: Case Report: Hemophagocytic Lymphocytosis in a Patient With Glutaric Aciduria Type IIC
Source: Front Immunol. 2022 Jan 13;12:810677. doi: 10.3389/fimmu.2021.810677 (PMC8792439; doi:10.3389/fimmu.2021.810677)

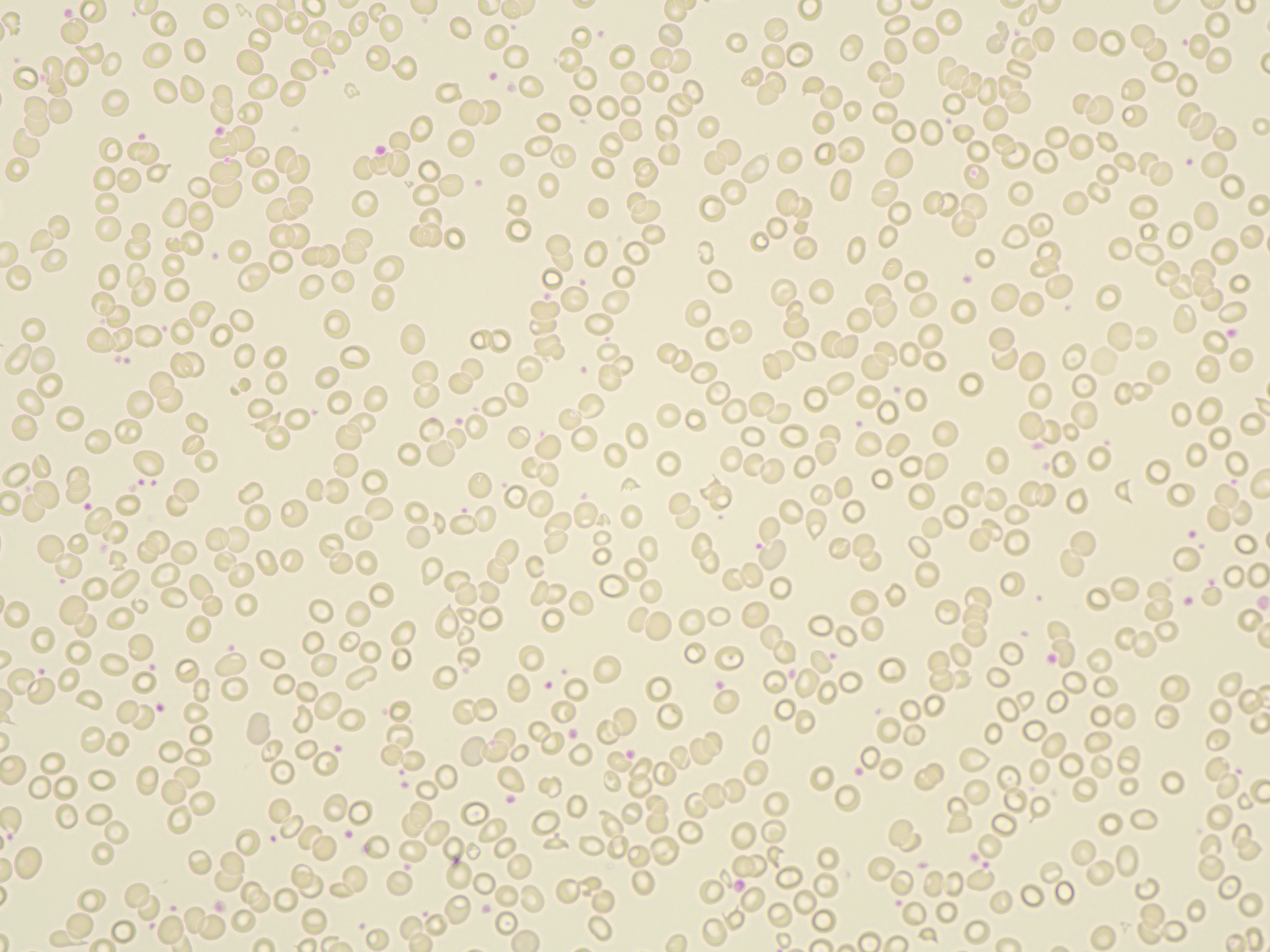

Supplement: Supplementary file 1 [file Image_1.tif]
